# *Demodex crocidurae*, a New Demodecid Mite (Acariformes: Prostigmata) Parasitizing the Lesser White-Toothed Shrew and a Redescription of *Demodex talpae* from European Mole with Data on Parasitism in Soricomorpha

**DOI:** 10.3390/ani11092712

**Published:** 2021-09-17

**Authors:** Karolina Cierocka, Joanna N. Izdebska, Leszek Rolbiecki

**Affiliations:** Department of Invertebrate Zoology and Parasitology, Faculty of Biology, University of Gdańsk, Wita Stwosza 59, 80-308 Gdańsk, Poland; karolina.cierocka@ug.edu.pl (K.C.); leszek.rolbiecki@ug.edu.pl (L.R.)

**Keywords:** Acariformes, Prostigmata, Demodecidae, parasite, skin mites, mammals, Soricidae, Talpidae

## Abstract

**Simple Summary:**

This paper describes a new species, *Demodex crocidurae*, inhabiting the hairy skin of Crocidura suaveolens. It also redescribes the most morphologically similar form: *Demodex talpae* from Talpa europaea, a species known from Hirst’s 1921 description. Following a differential diagnosis, it was concluded that these are separate species with different features important in the taxonomy of Demodecidae, inhabiting analogous microhabitats in different species of hosts.

**Abstract:**

Only six parasitic species of Demodecidae mite have thus far been described from the Soricomorpha, these being associated with the common shrew *Sorex araneus* Linnaeus, 1758, and the Mediterranean water shrew *Neomys anomalus* Cabrera, 1907 (two species from each host), and with the lesser white-toothed shrew *Crocidura suaveolens* (Pallas, 1811) and the European mole *Talpa europaea* Linnaeus, 1758 (one from each host species). Presently, *Demodex crocidurae*, a new species, has been described from the territory of Poland for *C. suaveolens*; in order to confirm its validity, it was necessary to redescribe *D. talpae* Hirst, 1921, from *T. europaea*, a demodecid species first described by Hirst in 1921 from England and then noted only in Poland. Both species colonized the hairy skin of the body in their hosts, where no disease symptoms of infestation were observed. However, *D. crocidurae* showed higher infection parameters (prevalence 100%, mean intensity 11.7, intensity range 3–26 individuals) than those of *D. talpae* (30.0%, 4.7, 2.0–8.0), possibly due to different host biology.

## 1. Introduction

The Demodecidae (Acariformes: Prostigmata) fauna of the soricomorphs (Soricomorpha) has been poorly studied. Six species have been described in this group, and only 11 original publications are known worldwide [1]. The majority (eight) of these records concern the Mediterranean water shrew *Neomys anomalus* Cabrera, 1907, and the common shrew *Sorex araneus* Linnaeus, 1758; for each of these hosts, two Demodecidae species were described [2,3,4,5,6]. Thus far, only one species, *Demodex foveolator* Bukva, 1984, has been known from the lesser white-toothed shrew *Crocidura suaveolens* (Pallas, 1811), found in this host only in the tail region. It was described from the Czechia and recently recorded from Poland [7,8]. In contrast, *Demodex talpae* Hirst, 1921, collected from the European mole *Talpa europaea* Linnaeus, 1758, was described based on several specimens obtained from a single mole specimen from the United Kingdom [9] and was mentioned in a brief report from Poland covering the parasite fauna of the mole [10].

However, it appears that these data do not reflect the actual distribution of the Demodecidae in the Soricomorpha, a group comprising approx. 500 mammal species, several of which are widely distributed. The common shrew, Mediterranean water shrew, lesser white-toothed shrew, and the European mole mentioned above, exhibit distributions covering the entirety of Europe and the Palearctic [11,12]. The absence of data regarding the presence of Demodecidae in these host species likely stems from the lack of appropriate studies, as this group is rarely mentioned in comprehensive analyses of parasitofauna communities. However, the generic diversity of these mites in the soricomorphs is interesting because apart from the most commonly noted and the most species-rich *Demodex*, the genera *Apodemodex* and *Soricidex* have been described only from Soricomorpha [1,2,5,13].

Recently, a new *Demodex* species has been found in the hairy skin of the body of the lesser white-toothed shrew. In terms of morphological traits, it was found to be similar to *D. talpae*, which was previously known from a highly laconic description by Hirst [9], supplemented with an illustration. Therefore, in order to verify the status of the presently discovered demodecid from the lesser white-toothed shrew, a redescription *D. talpae* was necessary.

## 2. Materials and Methods

Eight specimens of dead *C. suaveolens* from Poland (Wielkopolska Voivodeship, Słomowo, 52°21′11″ N, 17°32′41″ E) and ten *T. europaea* (Wielkopolska Voivodeship, Słomowo, 52°21′11″ N, 17°32′41″ E, six moles; Pomeranian Voivodeship, Pszczółki, 54°10′21″ N, 18°41′54″ E, three moles; Lublewko 54°45′28″ N, 17°55′37″ E, one mole), collected from October 2016–May 2019, were examined for demodecid mites.

Demodecidae were isolated using the digestion method developed for the detection of mammalian skin mites [14], with modifications to suit the examined host. Skin fragments of 1 cm^2^ were excised with a scalpel from several body regions, including the head (around eyes, ear pinnae, nose, area of vibrissae, lips, chin, cheeks, vertex), neck, abdomen, back, limbs, tail, and the genital–anal area. Skin samples were preserved in 70% ethanol and subjected to digestion in 10% potassium hydroxide solution until it was completely digested. The obtained samples were decanted (examination of 1 cm^2^ of the skin equal to the analysis of approximately 100 wet preparations), mounted and examined using phase–contrast microscopy (Nikon Eclipse 50 i). The mites were placed in polyvinyl-lactophenol solution and measured (measurements in micrometers) as follows: total body length = length of gnathosoma, podosoma and opisthosoma; gnathosomal width = width at base; podosomal and opisthosomal width = maximum width.

The specimen depositories are cited using the following abbreviation: UGDIZP, University of Gdańsk, Department of Invertebrate Zoology and Parasitology, Gdańsk, Poland. The description of the species adopted the nomenclature commonly used for the family Demodecidae [15] and was completed with the nomenclature proposed by Bochkov [16] for the superfamily Cheyletoidea (Acariformes: Prostigmata) and by Izdebska and Rolbiecki [17]. The scientific and common names of the hosts follow Wilson and Reeder [11] and the Taxonomic Information System [18].

To define the level of host infection, the following main parasitological parameters were measured: prevalence (percentage of hosts infected), mean intensity (mean number of parasites in infected hosts), and intensity range (minimum and maximum number of parasite individuals per host) [19].

## 3. Results

### 3.1. Demodex crocidurae Izdebska, Cierocka, Rolbiecki, 2021

**Figure 1:**
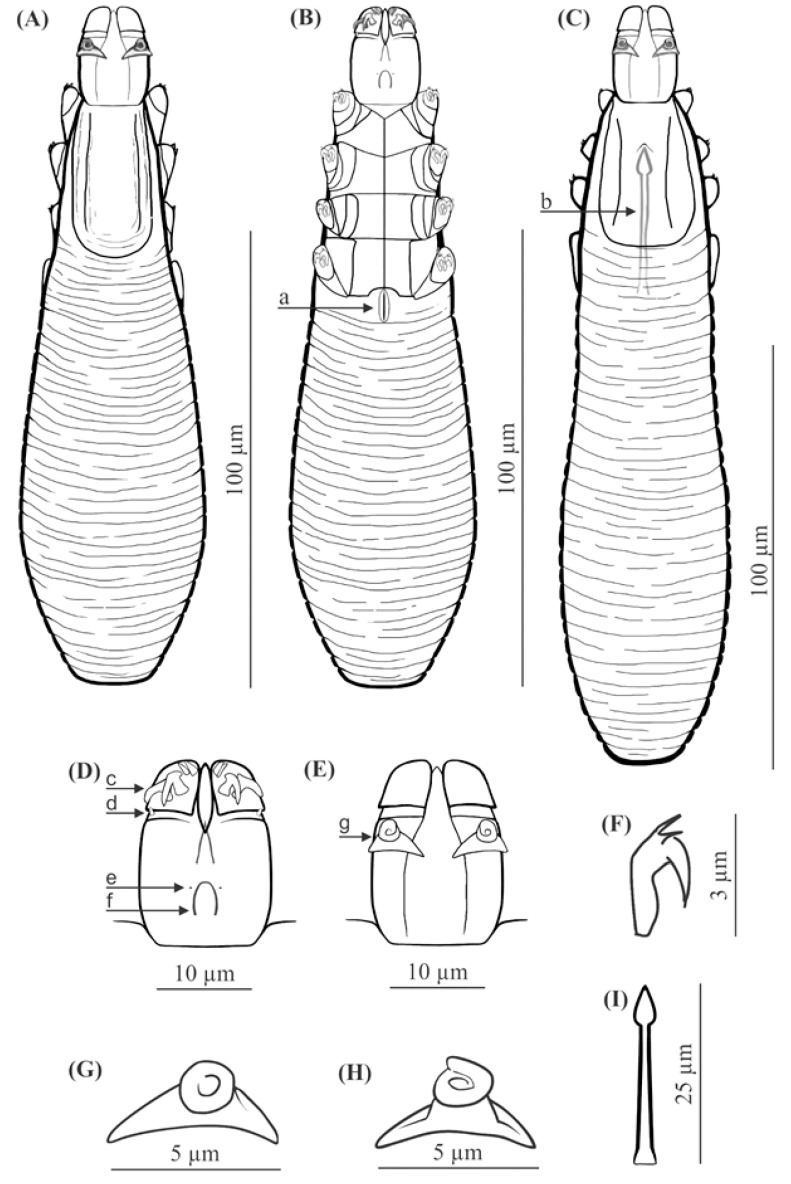
*Demodex crocidurae* sp. nov.: female, dorsal view (**A**), female, ventral view (**B**), male, dorsal view (**C**), gnathosoma, female, ventral view (**D**), gnathosoma, female, dorsal view (**E**), claw on the leg (**F**), supracoxal spine, dorsal view (**G**), supracoxal spine, lateral view (**H**), aedeagus (**I**); a: vulva, b: aedeagus, c: spines on palps, d: seta *v”F*, e: subgnathosomal seta (seta *n*), f: pharyngeal bulb, g: supracoxal spine (seta *elc.p*).

**Figure 2:**
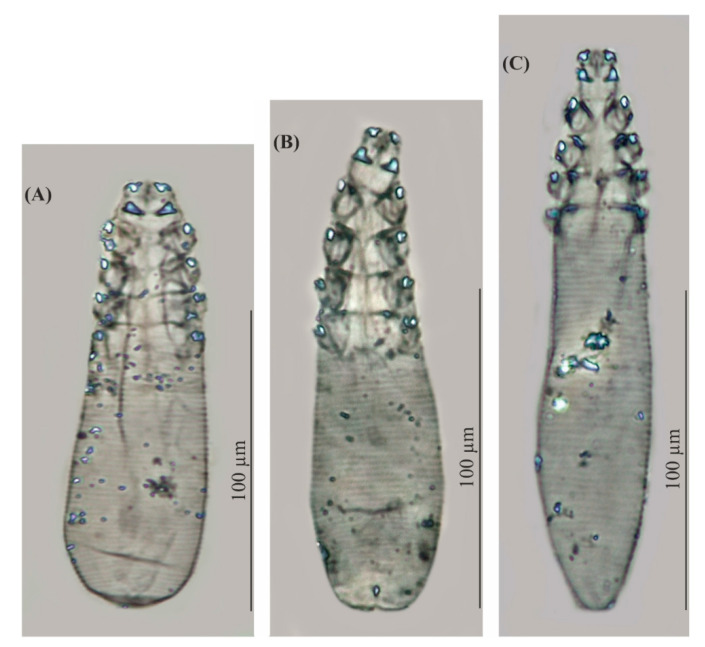
*Demodex talpae*, female (**A**) and *Demodex crocidurae* sp. nov., female, holotype (**B**), male (**C**).

Description (Table 1, Figure 1 and Figure 2)

FEMALE (holotype and 60 paratypes): Body 153 (138–176) long and 29 (23–35) wide (holotype, 150 × 27). Distinctly separated gnathosoma, rectangular, longer than wide; on dorsal surface in anterior part of basal (coxal) segment, parallel to base, pair of massive boomerang-shaped supracoxal spines (setae *elc.p*) present, ca. 5.0 long (holotype, 5.0). Palps 3-segmented, terminating in two claw-like, large spines on tibio-tarsus, ca. 2.0 long (holotype, 2.0) and two small, conical spines; conical setae *v″F* near external edge of middle segment (trochanter-femur-genu) present. On ventral surface, horseshoe-shaped pharyngeal bulb with pair of conical subgnathosomal setae (setae *n*) situated on either side of and slightly posterior to anterior limit. Podosoma trapezoidal, widening posterior end; four pairs of short legs, with coxae integrated into ventral idiosomal wall and five free, overlapping segments (trochanter–tarsus); two forked claws, ca. 3.0 long (holotype, 3.0) with large, pointed subterminal spur, and one solenidion (*ω*) on each tarsus. Epimeral plates (coxal fields) distinctly sclerotized; pairs I-II trapezoidal, III-IV pairs rectangular; posterior edges of pair IV indented archwise; anterior end of vulva between incision. Podosomal shield reaching level of legs III; posterior edge of this shield is convex. Opisthosoma constitutes 57% (53−63%) of body length (holotype, 57%); widens to 3/4 of opisthosoma length and then becomes narrower. Whole opisthosoma distinctly annulated; annulation reaches level of legs III dorsally; annuli relatively wide, ca. 1.5−2.0 µm. Opisthosomal organ absent. Vulva 6 (5−10) long (holotype, 8.0), located between and behind incision of IV epimeral plate.

MALE (32 paratypes): Slightly longer and slender than female, 158 (139−182) long, 30 (24−35) wide. Distinctly separated gnathosoma, rectangular, longer than wide. Pharyngeal bulb and morphological details of gnathosoma similar to those in females. In addition, podosoma and legs shaped similar to those in females; however, posterior edge of epimeral plate IV lacks incision. Opisthosoma constitutes 61% (56−66%) of body length; opisthosoma, similar to females, distinctly annulated; annuli relatively wide, ca. 1.5−2.0 µm. Opisthosomal organ absent. Aedeagus 25 (22−30) long, on dorsal surface, located between epimeral plates II and IV. Genital opening located on dorsal surface, at level of anterior margin of epimeral plate II.

Type material: Female holotype (reg. no. UGDIZPSCSDDc13f) from *Crocidura suaveolens* (reg. no. MSSCs01/2017), Słomowo, Wielkopolska Voivodeship, Poland, August 2017, parasites coll. K. Cierocka, J.N. Izdebska, host coll. J.N. Izdebska, L. Rolbiecki; 60 female paratypes (reg. nos. UGDIZPSCSDDc01-12f, UGDIZPSCSDDc14-61f) and 32 male paratypes (reg. nos. UGDIZPSCSDDc01-32m) from *Crocidura suaveolens* (reg. nos. MSSCs01/2017, MSSCs02/2018, MSSCs03-05/2017, MSSCs06-08/2018), Słomowo, Wielkopolska Voivodeship, Poland, August 2017, August 2018, same collectors.

Type material deposition: Whole type material (mounted microscope slides with the demodecid mites) is deposited in scientific collections within the framework of the Collection of Extant Invertebrates in Department of Invertebrate Zoology and Parasitology, University of Gdańsk, Poland.

Infection and location in the host. *Demodex crocidurae* sp. nov. was found in all examined lesser white-toothed shrews (100%), with a mean intensity of 11.7 and intensity range of 3–26 individuals per host: 93 individuals in total (32 males, 61 females). The demodecid mites were found on the hairy skin of the body (head—31 individuals, abdomen—30, back—28, and genital-anal area—4). The observed mites did not cause any lesions in examined shrews.

Etymology. The specific epithet *crocidurae* refers to the specific name of the host.

### 3.2. Demodex talpae Hirst, 1921

**Figure 3:**
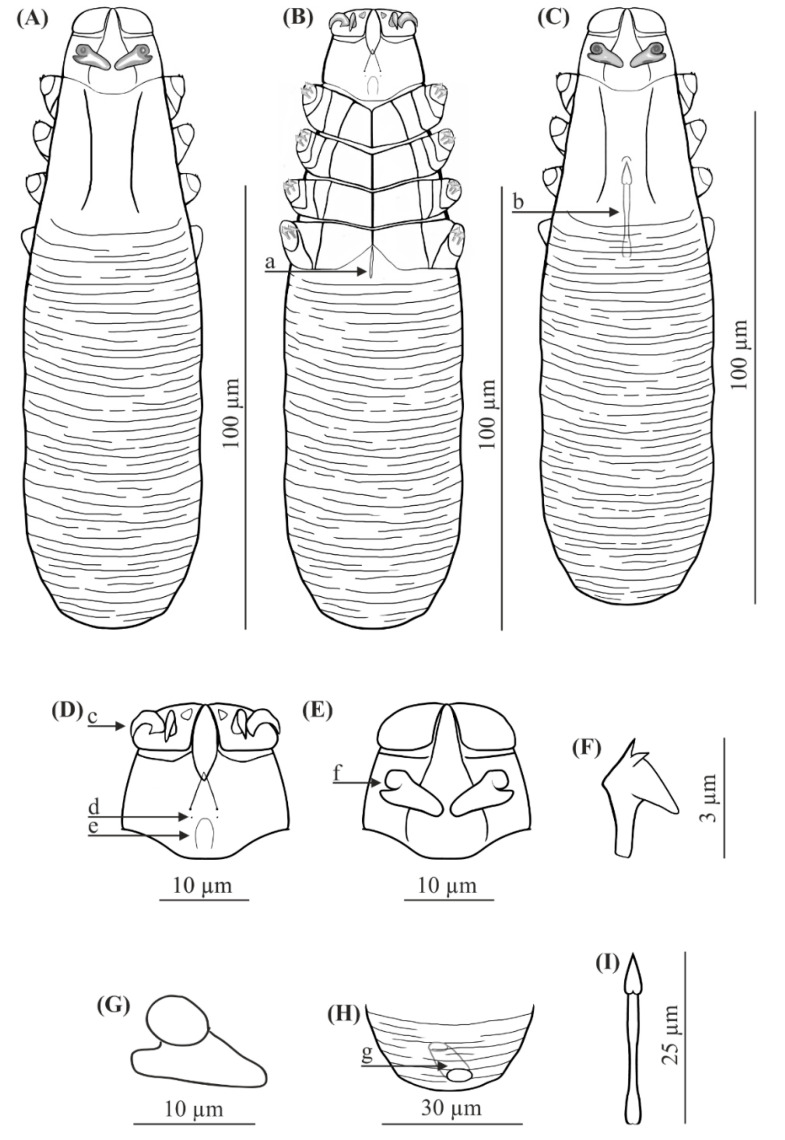
*Demodex talpae*: female, dorsal view (**A**), female, ventral view (**B**), male, dorsal view (**C**), gnathosoma, female, ventral view (**D**), gnathosoma, female, dorsal view (**E**), claw on the leg (**F**), supracoxal spine (**G**), opisthosomal organ, female (**H**), aedeagus (**I**); a: vulva, b: aedeagus, c: spines on palps, d: subgnathosomal seta (seta *n*), e: pharyngeal bulb, f: supracoxal spine (seta *elc.p*), g: opisthosomal organ.

Redescriptions (Table 2, Figure 2 and Figure 3)

FEMALE (*n* = 8): Body 143 (134–154) long and 38 (35–43) wide. Distinctly separated gnathosoma, trapezoidal, base width longer than length; on dorsal surface in anterior part of basal (coxal) segment, directed posteromedially, pair of massive, wedge-shaped supracoxal spines (setae *elc.p*) present, ca. 5.5 long. Palps 3-segmented, terminating in two claw-like, large spines on tibio-tarsus, ca. 3.0 long and one small, conical spine. On ventral surface, horseshoe-shaped pharyngeal bulb with pair of conical subgnathosomal setae (setae *n*) situated exactly at level of anterior margin on both sides. Podosoma trapezoidal, widening posterior end; four pairs of short legs, with coxae integrated into ventral idiosomal wall and five free, overlapping segments (trochanter–tarsus); two forked claws, ca. 3.0 long with large, pointed subterminal spur. Epimeral plates (coxal fields) distinctly sclerotized; I-IV pairs trapezoidal; posterior edges of pair IV weakly sclerotized, form triangular incision, which almost surrounding vulva. Podosomal shield reaching level of legs III; posterior edge of this shield is concave. Opisthosoma constitutes 57% (53−60%) of body length; widens towards end; widest at end. Opisthosoma distinctly and densely annulated; annulation also reaches dorsal podosoma side (level of legs III); annuli relatively wide at *ca.* 1.0–1.5 µm. Opisthosomal organ present, tubular; opisthosomal pore oval, 1.5–2.0 in diameter. Vulva 9 (8−10) long, located between and behind incision of IV epimeral plate.

MALE (*n* = 6): Shorter than female, 128 (118−150) long, 34 (33−40) wide. Distinctly separated gnathosoma, trapezoidal, base width longer than length. Pharyngeal bulb and morphological details of gnathosoma similar to those in female. Shape of podosoma and legs similar to those in female; however, only posterior edge of epimeral plate IV without incision. Opisthosoma constitutes 58% (55−60%) of body length; widens towards posterior end. Opisthosoma, similar to females, distinctly annulated; annuli relatively wide, ca. 1.0−1.5 µm. Opisthosomal organ present, similar to that in female. Aedeagus 19 (18−22) long, on dorsal surface, located between epimeral plates III and IV. Genital opening located on dorsal surface, on border between epimeral plates II and III.

Material deposition: Mounted microscope slides with specimens were stored in scientific collections within the framework of the Collection of Extant Invertebrates in Department of Invertebrate Zoology and Parasitology, University of Gdańsk, Poland. Eight females (reg. no. UGDIZPTTeDDt01-08f) and six males (reg. no. UGDIZPTTeDDt01-06m) from *Talpa europaea* (reg. no. MSTTe01/2016, MSTTe02/2019, MSTTe03/2018), Lublewko, Pomeranian Voivodeship, Słomowo, Wielkopolska Voivodeship, Pszczółki, Pomeranian Voivodeship, Poland, October 2016, August 2018, May 2019, parasites coll. K. Cierocka, J.N. Izdebska, host coll. J.N. Izdebska, L. Rolbiecki.

Infection and location in the host. *Demodex talpae* was noted in 30.0% of the ten examined European moles, with a mean intensity of 4.7 and intensity range of 2.0–8.0 individuals per host: 14 individuals in total (six males, eight females). Mites were found in the hairy skin of the body (back—six individuals; abdomen—eight). The observed mites did not cause any lesions in the examined European moles. Infestations were not associated with skin lesions or other symptoms.

### 3.3. Differential Diagnosis

Regarding the known Demodecidae, *D. crocidurae* sp. nov. appears to be closely related to *D. talpae* described from the European mole, this being a representative of another family within the order Soricomorpha. However, *D. talpae* is smaller and has different body proportions; it is relatively wider, with shorter opisthosoma; in addition, *D. talpae* males and females have similar body size and proportions, whereas *D. crocidurae* males are typically slightly longer (with longer opisthosoma) than the females (Table 1, Table 2 and Table 3, Figure 1, Figure 2 and Figure 3).

The gnathosoma in *D. crocidurae* is rectangular, narrow, and longer than wide; in *D. talpae*, it is trapezoidal, shorter than wide at the base. The supracoxal spines in both species are large and massive (ca. 5.0 um in length); however, they are boomerang shaped in *D. crocidurae* and wedge-shaped in *D. talpae*. Additionally, they are directed parallel to the base of the gnathosoma in *D. crocidurae* and are directed posteromedially in *D. talpae*. In addition, there is a process on the supracoxal spines; this is located in the center of the spine in *D. crocidurae* and at the widest part in *D. talpae*. The terminal segments of the palpi are equipped with two large and two small spines in *D. cocidurae*, while there are two large and one small spines in *D. talpae*. The subgnathosomal setae are located on either side of and slightly posterior to the anterior margin of the pharyngeal bulb in *D. crocidurae*, and exactly at the level of anterior margin of the pharyngeal bulb in *D. talpae*. The leg tarsi are equipped with forked claws differing in shape between both demodecid species. Differences also exist between the epimeral plates: in *D. crocidurae*, I–II pairs are trapezoidal and III–IV are rectangular, while in *D. talpae*, all epimeral plates are trapezoidal. Moreover, the posterior edge of epimeral plates IV has an arched shape in *D. crocidurae* females but a triangular shape in *D. talpae* females. In addition, the posterior edge of the podosomal shield is convex in *D. crocidurae* and concave in *D. talpae*. The opisthosoma in *D. crocidurae* is narrower and longer, widens to 3/4 of its length and then narrows, while in *D. talpae* it is wider and shorter, widening posteriorly, with the widest part at the end of the body. In addition, the opisthosomal organ is absent in *D. crocidurae*, but it is present in both sexes in *D. talpae*. Furthermore, in *D. crocidurae* males, the aedeagus is longer (22–30 μm in length) and located at epimeral plates II–IV with the genital opening at the level of anterior margin of epimeral plates II; in *D. talpae* males, the aedeagus is shorter (18–22 μm in length) and located at the border between epimeral plates III and IV, with the genital opening on the border between epimeral plates II and III.

## 4. Discussion

*Demodex crocidurae* sp. nov. clearly differs from all known Demodecidae species. However, it resembles *D. talpae* in general body shape, and certain characteristics, namely, the shape of the supracoxal spines and leg claws, which are also observed in several demodecid mites of the Muridae, e.g., *D. musculi* Oudemans, 1897, *D. apodemi* Hirst, 1918, *D. corniculatus* Izdebska, 2012, *D. bandicotae* Izdebska, Rolbiecki, Morand & Ribas, 2017 [20,21,22,23]. Although both the two host species, under discussion, belongs to the order Soricomorpha, they are representatives of two separate families, Soricidae and Talpidae, respectively with a doubtful level of affinity [11]. In addition, the lesser white-toothed shrew and the European mole have different environmental preferences and different biologies. *Crocidura suaveolens* is a small mammal (weight in the 3–7 g range), found in Central Europe, Israel, Saudi Arabia, Middle East, Caucasus, Kyrgyzstan, north-east China and Korea, where it primarily inhabits bushes, forest communities, as well as parks and gardens and buildings [24]. It forms a nest among grass or in abandoned rodent burrows. It is active throughout the year and day and night and has short activity and rest cycles. Conversely, *T. europaea* is considerably larger (up to 120 g) and has a considerably wider distribution range, including the Northern Hemisphere, being found in Europe, Asia and North America. It is also active throughout the year during both day and night, but it has longer activity and rest cycles. The mole is also a subterranean mammal, constructing underground nests and tunnels [12,25]. Thus, it is difficult to expect that the same Demodecidae species will be found in both host species.

*Demodex crocidurae* and *D. talpae*, although morphologically similar (Table 1, Table 2 and Table 3, Figure 1, Figure 2 and Figure 3) and found in comparable microhabitats on their hosts (hairy skin), were found to demonstrate different infestation levels. *Demodex crocidurae* was common in all examined shrews, despite originating from different populations and seasons. In contrast, *D. talpae*, demonstrates low infestation parameters; only 14 demodecid mites were recorded in only three mole specimens in the present study (30.0% of the examined).

Previously, *D. talpae* was only known from a single record from the United Kingdom, in which Hirst [9] described the species based on several specimens obtained from a single mole in 1919. However, *D. talpae* was recorded from several hosts in recent studies on the mole parasitofauna conducted in Poland [10]; but, due to the different study methodology (determined by parasite analyses from different groups and locations), and poor preservation state of the specimens, it was not possible to use all specimens for the redescription.

Knowledge of the distribution of *Demodex* species is still fragmentary the distribution of both species may be wider than presently known. In the present study, *D. crocidurae* was found in lesser white-toothed shrews from an area located near the northwestern boundary of their distribution in Europe; the shrew is found relatively rarely in this area, and considering its solitary behavior (apart from the breeding season), this likely does not facilitate the spread of this parasite. Despite this, *D. crocidurae* was well represented on all individuals of all the host populations. The considerably lower *D. talpae* infestation level observed in European mole is probably linked to its subterranean and extremely solitary behavior. However, its records from distant localities (the United Kingdom and Poland) over the period of 100 years clearly suggests that it may be present in more areas of its host distribution.

## 5. Conclusions

To date only six species (*Apodemodex cornutus* Bukva, 1996, *D. foveolator, D. neomydis* Bukva, 1995, *D. soricinus* Hirst, 1918, *D. talpae, Soricidex dimorphus* Bukva, 1982) of Demodecidae parasitic mites are recorded in the Soricomorpha. Here, we describe the seventh species, *D. crocidurae* from *C. suaveolens* which indicates that the diversity of these mites in this most primitive group of placental mammals is probably greater and requires further detailed research.

## Figures and Tables

**Table 1:** Measurements of morphological features (in micrometers) for adults of *Demodex crocidurae* sp. nov.

Morphologic Features	Males (*n* = 32)	Females (*n* = 61)
Length of gnathosoma	17 (15–20), SD 1	17 (15–20), SD 1
Width of gnathosoma (at base)	12 (11–16), SD 1	13 (10–16), SD 1
Length of podosoma	45 (40–53), SD 3	49 (40–55), SD 3
Width of podosoma	23 (19–28), SD 2	24 (20–30), SD 2
Length of opisthosoma	96 (80–120), SD 10	87 (73–110), SD 9
Width of opisthosoma	30 (24–35), SD 3	29 (23–35), SD 3
Aedeagus	25 (22–30), SD 2	–
Vulva	–	6 (5–10), SD 1
Total body length	158 (139–182), SD 11	153 (138–176), SD 9

**Table 2:** Measurements of morphological features (in micrometers) for adults of *Demodex talpae*.

Morphologic Features	Males (*n* = 6)	Females (*n* = 8)
Length of gnathosoma	13 (10–15), SD 2	14 (13–15), SD 1
Width of gnathosoma (at base)	18 (17–20), SD 1	18 (17–21), SD 2
Length of podosoma	42 (38–50), SD 6	48 (45–53), SD 3
Width of podosoma	30 (27–33), SD 2	33 (30–38), SD 3
Length of opisthosoma	74 (68–88), SD 7	81 (73–88), SD 6
Width of opisthosoma	34 (33–40), SD 3	38 (35–43), SD 3
Aedeagus	19 (18–22), SD 2	–
Vulva	–	9 (8–10), SD 1
Total body length	128 (118–150), SD 13	143 (134–154), SD 6

**Table 3:** Morphometric comparison between *Demodex crocidurae* sp. nov. and *Demodex talpae*.

Feature/Species	*Demodex crocidurae* sp. nov.		*Demodex talpae*			
Source	Present Study		Present Study		Hirst [9]	
Sex	Males	Females	Males	Females	Males	Females
Sample Size	(*n* = 32)	(*n* = 61)	(*n* = 6)	(*n* = 8)	(*n* = *)	(*n* = *)
Body total length	158 (139–182), SD 11	153 (138–176), SD 9	128 (118–150), SD 13	143 (134–154), SD 6	126	128–130
Body total width	30 (24–35), SD 3	29 (23–35), SD 3	34 (33–40), SD 3	38 (35–43), SD 3	37	34–41
Body length to width ratio	5.3:1 (4.3–6.1:1), SD 0.5:1	5.4:1 (4.3–7.0:1), SD 0.6:1	3.7:1 (3.5–3.9:1), SD 0.1:1	3.8:1 (3.2–4.1:1), SD 0.3:1	3.4:1 **	
Opisthosoma length to body length ratio (%)	61 (56–66), SD 3	57 (53–63), SD 3	58 (55–60), SD 2	57 (53–60), SD 2	57 **	
Aedeagus length	25 (22–30), SD 2	–	19 (18–22), SD 2	–	22	–
Vulva length	–	6 (5–10), SD 1	–	9 (8–10), SD 1	–	–

* The author gives no information about the number of examined mites; only “several specimens” are given. ** Taken from measurements of Hirst [9].
